# ESR study of development of RFM/Un murine myeloid leukaemia.

**DOI:** 10.1038/bjc.1978.257

**Published:** 1978-11

**Authors:** N. J. Dodd, J. M. Silcock

## Abstract

The blood, spleen and liver of RFM/Un mice were examined by means of electron spin resonance (ESR) throughout the course of myeloid leukaemia induced by i.v. injection of leukaemic spleen cells. Marked changes in the concentration of iron transferrin and caeruloplasmin were observed in the blood 1 day after injection. As the disease progressed, changes occurred in the concentrations of the ascorbyl radical and of paramagnetic metal complexes in both spleen and liver. These changes are compared with those induced in RF/J mice injected with normal and leukaemic spleen cells from RFM/Un mice.


*Br. J. Cancer* (1978) **38**, 612

### ESR STUDY OF DEVELOPMENT OF RFM/Un MURINE MYELOID LEUKAEMIA

N. J. F. DODD AND J. M. SILCOCK

*From the Paterson Laboratories, Christie Hospital and Holt Radium Institute, Manchester M20 9BX*

Received 21 June 1978 Accepted 11 August 1978

**Summary.—The blood, spleen and liver of RFM/Un mice were examined by means of electron spin resonance (ESR) throughout the course of myeloid leukaemia induced by i.v. injection of leukaemic spleen cells. Marked changes in the concentration of iron transferrin and caeruloplasmin were observed in the blood 1 day after injection. As the disease progressed, changes occurred in the concentrations of the ascorbyl radical and of paramagnetic metal complexes in both spleen and liver. These changes are compared with those induced in RF/J mice injected with normal and leukaemic spleen cells from RFM/Un mice.**

ELECTRON SPIN RESONANCE (ESR) has been used to study changes in the concentration of radicals or paramagnetic metals in lyophilized, fresh and frozen tissues (see Mallard & Kent, 1969; Swartz, 1972). More recently, experiments have examined the changes in the concentration of paramagnetic metal ions (Swartz *et al.*, 1973; Dodd, 1975; Dodd & Silcock, 1976) and a particular free radical, the ascorbyl radical (Dodd, 1973; Dodd & Giron-Conland, 1975; Giron-Conland, 1975; Silcock & Dodd, 1976) during the development of experimental malignancies in mice and rats. This paper reports ESR studies on a murine myeloid leukaemia carried in RFM/Un mice, and compares the results with those obtained in RF/J mice.

### MATERIALS AND METHODS

The RFM/Un leukaemia was induced by radiation in the Biology Division, Oak Ridge National Laboratory, Tennessee. The line used in the experiments described here was obtained from the Radiobiology Department, The Medical College of St Bartholomew's Hospital, London, in 1973. The disease is passaged by the i.v. injection of $\sim 10^6$ leukaemic spleen cells, and is terminal at 7–8 days.

RFM/Un male mice aged $\sim 3$ months were used. The experimental animals were injected with $\sim 10^6$ leukaemic spleen cells and the controls with $\sim 10^6$ normal spleen cells and examined over the 8-day period. The animals were starved overnight before being killed. Samples of blood were taken by cardiac puncture whilst the animals were under ether narcosis, and the spleen and left lobe of the liver were then removed. The spleen weight was recorded and used as a measure of the progression of the disease.

ESR measurements were made with a Varian E-9 X-band spectrometer. Tissue samples weighing $\sim$10 mg were examined for the presence of the ascorbyl radical, at room temperature, in a flat quartz cell. The accurate weight of the tissue sample was recorded. About 5 animals were examined for each experimental point. The spectrometer was operated, in conjunction with a Nicolet 1020A signal averager, at conditions previously described (Dodd & Giron-Conland, 1975; Silcock & Dodd, 1976). The spectra were quantitated by recording the relative heights of the ascorbyl-radical signal and the manganese standard signal. Tissue spectra were corrected for the weight of the sample and the results were expressed as the relative signal height per g of tissue. Samples of blood, spleen and liver were examined at $-196$°C for the presence of paramagnetic metals. About 12 animals were examined for each experimental point. The methods used to prepare samples and record their spectra have been described previously (Dodd, 1975). Signal heights were measured relative to that of the manganese standard.

Parallel studies were carried out on the effects of injection of $\sim 10^6$ leukaemic spleen cells from an RFM/Un mouse into RF/J female mice aged $\sim 3$ months. The RFM/Un leukaemia does not take in RF/J mice. The effects of injecting $\sim 10^6$ normal spleen cells from RFM/Un into RF/J mice were also examined.

Student's $t$ test was used to calculate confidence limits.

### RESULTS

### *Histology*

The spleen looked normal on Day 1 of the disease, but by Day 4 leukaemic colonization could be seen. This became more extensive with time. The spleen weight increased rapidly after Days 4–5, reaching about 3 × normal weight by Day 8 (Fig. 1). Leukaemic infiltration of the liver could be detected histologically by Days 5–6. However, even at this late stage of the disease the colonies still seemed to be associated with the areas around the blood vessels.

Fig. 1.—Height of the ascorbyl-radical signal, relative to the manganese standard, per g of tissue, in slices taken from the middle of the spleen of RFM/Un male mice during development of a myeloid leukaemia. (Solid line). Vertical lines show the standard error of the experimental points. Dotted line shows the change in spleen weight with development of the disease.

Fig. 2.—Height of the ascorbyl-radical signal relative to the manganese standard per g of tissue, in slices taken from the middle of the spleen of RF/J female mice after an injection of cells from leukaemic spleens of RFM/Un mice. (Solid line). Vertical lines show the standard error of the experimental points. Dotted line shows the change in spleen weight with time after injection of leukaemic cells from the spleens of RFM/Un mice.

### *Ascorbyl radical*

The concentration of the ascorbyl radical in slices from the middle of the spleen and the hepatic-vein region of the liver was on the limits of detection. An increase in concentration occurred in the spleen after Day 6 of the disease ($P <$ 0·005) (Fig. 1). Little change was noted in the concentration of ascorbyl radical in the liver. No change was observed in the concentration of ascorbyl radical in the spleen or liver after injection of normal spleen cells from RFM/Un mice.

Injection of leukaemic spleen cells

FIG. 3.—Heights of ESR signals, relative to the manganese standard, in the blood of RFM/Un male mice, during the development of leukaemia. Vertical lines show the standard error of the experimental points.

from RFM/Un mice into RF/J animals induced an increase in the concentration of ascorbyl radical in the spleen on Days 2 and 3 ($P < 0{\cdot}005$) (Fig. 2). These resembled the changes observed during the development of the RF/J leukaemia (Dodd & Giron-Conland, 1975; Giron-Conland, 1975). The apparent increase in the concentration of ascorbyl radical in the spleens of RF/J mice on Days 2–4 after the injection of normal cells from RFM/Un mice was not significant ($P < 0{\cdot}3$).

*Paramagnetic metals ions*

*Blood*.—The major features of the ESR spectrum of blood are the characteristic 3-line spectrum of iron(III) bound to the serum protein transferrin at a $g$ value of 4·3, and the signal at $g \sim 2{\cdot}05$ from the copper(II) of caeruloplasmin. The concentration of iron transferrin in the blood of leukaemic RFM/Un mice showed

FIG. 4.—Heights of ESR signals, relative to the manganese standard, in the blood of RF/J female mice, after injection of cells from leukaemic spleens of RFM/Un mice. Vertical lines show the standard error of the experimental points.

a distinct biphasic change with time (Fig. 3). Iron transferrin increased to a maximum on Day 2, returned almost to normal by Days 4 and 5, and then increased sharply in the terminal stages of the disease. Caeruloplasmin was generally elevated throughout the disease, and appeared to be maximal on Days 3 and 7.

The initial changes in the blood of leukaemic RFM/Un mice were seen in the blood of RF/J mice injected with leukaemic spleen cells from RFM/Un mice, but the later changes were absent (Fig. 4). A smaller but similar initial change was seen in the blood of RF/J mice injected with normal cells from RFM/Un mice.

*Spleen.*—The ESR signals in frozen samples of spleen have been tentatively assigned to various complexes of paramagnetic metal ions (Dodd, 1975). Relative changes in the concentrations of these species during development of the disease are shown in Fig. 5a and b. The signal at $g \sim 6{\cdot}0$, due to high-spin haem

FIG. 5.—Heights of ESR signals, relative to the manganese standard, in the spleen of RFM/Un male mice, during the development of leukaemia, at $g$ values as shown. Vertical lines show the standard error of the experimental points.

iron(III), decreased slowly over the first 4 days of the disease, and then fell rapidly to zero. This change coincided with a rapid increase in spleen weight. The signal at $g \sim 4{\cdot}3$, due to high-spin, non-haem iron(III), appeared to increase to a maximum on Day 2 and then decrease slightly as the disease progressed. The signal at $g \sim 2{\cdot}04$ showed a slight decrease with time. This signal has not been identified, but may be a mixture of copper(II) or low-spin iron(III) complexes. However, it bears a resemblance to certain nitric oxide–haemoprotein derivatives, showing a maximum at $g$ of 2·075 (Henry & Banerjee, 1973). It may therefore be a normal product of haemoprotein catabolism. The signal at $g \sim 2{\cdot}00$, which is thought to be a flavin-semiquinone free radical, showed considerable variation with time, being maximal on about Day 3 and possibly just before death. An increase in the concentration of the $g \sim 1{\cdot}94$ signal, thought to be non-haem iron close to a sulphur atom, was observed in the later stages of the disease.

When RF/J mice were injected with RFM/Un leukaemic spleen cells, no significant changes were observed in the signals at $g$s of 2·04, 2·00 and 1·94. However the $g \sim 6$ signal showed a small decrease with time, while the $g \sim 4{\cdot}3$ signal increased slightly in the first 3 or 4 days after injection and then returned to normal. In these mice the normal level of the $g \sim 4{\cdot}3$ signal in the spleen is about 3 times that in the RFM/Un mice.

*Liver*.—The changes in the concentrations of signals at $g \sim 2{\cdot}25$, assigned to cytochrome $P_{450}$ (together with signals at 2·4 and 1·91), $g \sim 2{\cdot}00$, a flavin semiquinone free radical and $g \sim 1{\cdot}94$, a sulphur-containing low-spin iron complex, are shown in Fig. 6. These signals all showed minor fluctuations for the first 4 or 5 days after injection of leukaemic cells and then, as leukaemic infiltration of the liver became detectable, gradually decreased. The concentrations of the species with $g$ signals at values of 4·3,

FIG. 6.—Heights of ESR signals, relative to the manganese standard, in the liver of RFM/Un male mice, during the development of leukaemia, at $g$ values as shown. Vertical lines show the standard error of the experimental points.

2·05 and 1·97 showed little or no change during development of the disease. The signal at $g \sim 4{\cdot}3$ is from non-haem high-spin iron(III) and has recently been shown to be the water-insoluble storage iron compound, haemosiderin (van Leeuwen *et al.*, 1977). The water-soluble component of storage iron, ferritin, is not detectable at low concentration owing to its broad ESR signal (Boas & Troup, 1971). The signal at $g \sim 1{\cdot}97$ may be from the molybdohaemoprotein, sulphite oxidase (Peisach *et al.*, 1971), probably with contributions from low-spin iron compounds. The nature of the broad signal at $g \sim 2{\cdot}05$ is unknown. It may be a haemoprotein degradation product. The signal at $g \sim 2{\cdot}035$, assigned to a nitric oxide–iron, non-haem complex (Woolum *et al.*, 1968) appeared to be unchanged with time, but owing to its close proximity to the $g \sim 2{\cdot}00$ signal could not be measured accurately. The liver of RFM/Un mice, unlike that of RF/J, showed no detectable catalase signal.

### DISCUSSION

The concentration of the ascorbyl radical is thought to reflect the rate of oxidation of ascorbic acid rather than the amount of the acid in the tissues. Thus an increase in concentration of the radical during disease may reflect a change in the relative concentrations of ascorbic acid and oxidant caused by altered metabolism or cell lysis. These changes may be associated with a host–foreign cell or foreign cell–host response, as would appear to be the case with RF/J mice injected with cells from the RFM/Un strain, or with competitive cellular proliferation.

Changes in the concentration of paramagnetic species in the blood of RFM/Un mice developing myeloid leukaemia are similar to those observed in the blood of RF/J mice developing their myeloid leukaemia (Dodd, 1975). The time scale of these changes is consistent with the time scale of the disease. ESR measurement suggest two distinct processes in the blood. One, occurring within a few days of injection, is also observed in the blood of RF/J mice injected with RFM/Un leukaemic spleen cells. Small changes in the concentrations of iron transferrin and caeruloplasmin were also observed in the blood of RF/J mice after injection of normal RFM/Un spleen cells, but not after injection of isologous spleen cells. This initial response may be associated with a host–foreign cell and/or possibly a foreign cell–host response, and is not dependent on subsequent development of disease. The secondary changes occurring in the blood are only detected in the developing leukaemias and are probably associated with cellular breakdown and release of iron, which is then taken up by transferrin. The changes observed in the blood of the leukaemic mice are, however, quite different from those in the blood of rats after implantation of a solid Yoshida tumour (Dodd and Silcock, 1976), but are similar to the changes in the blood of rats with developing Yoshida ascites tumours (unpublished). In the case of the solid tumour, no change is detectable in the blood within the first 4 days of implantation owing, we believe, to an initial lack of adequate blood supply to the tumour. The ascites tumour, on the other hand, is induced by injection into, and is present in the peritoneal cavity, consequently there is no necessity for anastomosis.

The early changes in concentrations of paramagnetic species in spleen and liver of leukaemic RFM/Un mice are ill-defined, but may be associated with metabolic disturbance owing to the presence of foreign cells. The major changes are observed only when leukaemic infiltration of these organs is histologically detectable, changes in spleen being seen before those in liver. Similar changes are not seen in the spleens of RF/J mice injected with RFM/Un leukaemic cells, where leukaemia does not develop. It is thought that the changes may be associated with a general breakdown in normal metabolic activity and cell destruction caused by the leukaemic infiltration.

The authors would like to thank Dr O. G. Dodge, Consultant Pathologist, Christie Hospital, for his interpretation of the histological data, and Mr R. W. Thompson for injecting the animals. The work was supported by the Medical Research Council and the Cancer Research Campaign and one of the authors (J.M.S.) was holder of an MRC postgraduate training award.

### REFERENCES

Boas, J. F. & Troup, G. J. (1971) Electron spin resonance and Mössbauer effect studies of ferritin. *Biochim. Biophys. Acta*, **229**, 68.

Dodd, N. J. F. (1973) Some EPR signals in tumour tissue. *Br. J. Cancer*, **28**, 257.

DODD, N. J. F. (1975) Electron spin resonance study of changes during the development of a mouse myeloid leukaemia, I. The paramagnetic metal ions. *Br. J. Cancer*, **32**, 108.

DODD, N. J. F. & GIRON-CONLAND, J. M. (1975) Electron spin resonance study of changes during the development of a mouse myeloid leukaemia, II. The ascorbyl radical. *Br. J. Cancer*, **32**, 451.

DODD, N. J. F. & SILCOCK, J. M. (1976) Electron spin resonance study of changes during development of solid Yoshida tumour. II. Paramagnetic metal ions. *Br. J. Cancer*, **34**, 556.

GIRON-CONLAND, J. M. (1975) Electron spin resonance studies of the changes in the ascorbyl radical concentration in rat and mouse tissues during experimental malignancies. *Ph.D. Thesis, University of Manchester.*

HENRY, Y. & BANERJEE, R. (1973) Electron paramagnetic studies of nitric oxide haemoglobin derivatives: Isolated subunits and nitric oxide hybrids. *J. Mol. Biol.*, **73**, 469.

MALLARD, J. R. & KENT, M. (1969) Electron spin resonance in biological tissues. *Phys. Med. Biol.*, **14**, 373.

PEISACH, J., OLTZIK, R. & BLUMBERG, W. E. (1971) The electron paramagnetic resonance of molybdenum in rat liver and in rat liver mitochondria. *Biochim. Biophys. Acta*, **253**, 58.

SILCOCK, J. M. & DODD, N. J. F. (1976) Electron spin resonance study of changes during development of solid Yoshida tumour. I. Ascorbyl radical. *Br. J. Cancer*, **34**, 550.

SWARTZ, H. M. (1972) Cells and tissues. In *Biological Applications of Electron Spin Resonance*. Eds. H. M. Swartz, J. R. Bolton and D. C. Borg. New York: Wiley-Interscience. p. 155.

SWARTZ, H. M., MAILER, C., AMBEGAONKAR, S., ANTHOLINE, W. E., MCNELLIS, D. R. & SCHNELLER, S. J. (1973) Paramagnetic changes during development of a transplanted AKR/J leukaemia in mice as measured by electron spin resonance. *Cancer Res.*, **33**, 2588.

VAN LEEUWEN, F. X. R., ZUYDERHOUDT, F. J. M., VAN GELDER, B. F. & VAN GOOL, J. (1977) An EPR study of water-insoluble iron in human and rat liver. *Biochim. Biophys. Acta*, **500**, 304.

WOOLUM, J. C., TIEZZI, E. & COMMONER, B. (1968) Electron spin resonance of iron–nitric oxide complexes with amino acids, peptides and proteins. *Biochim. Biophys. Acta*, **160**, 311.


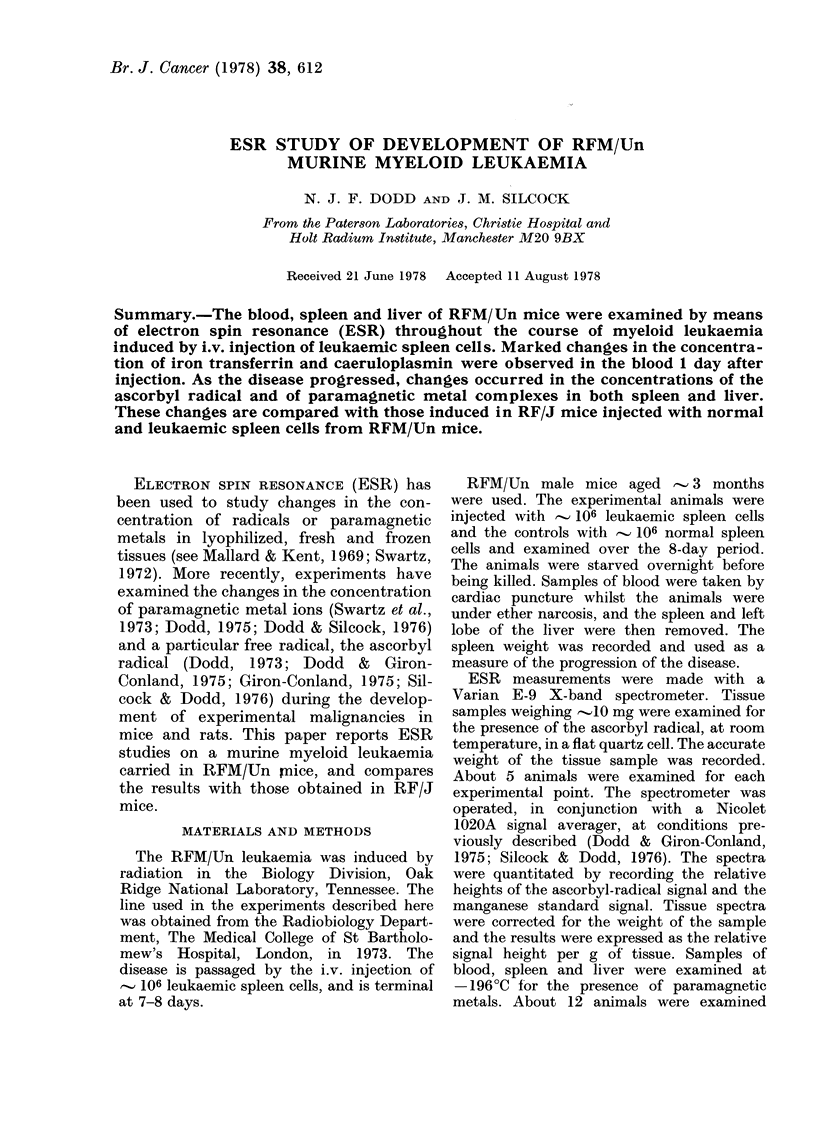

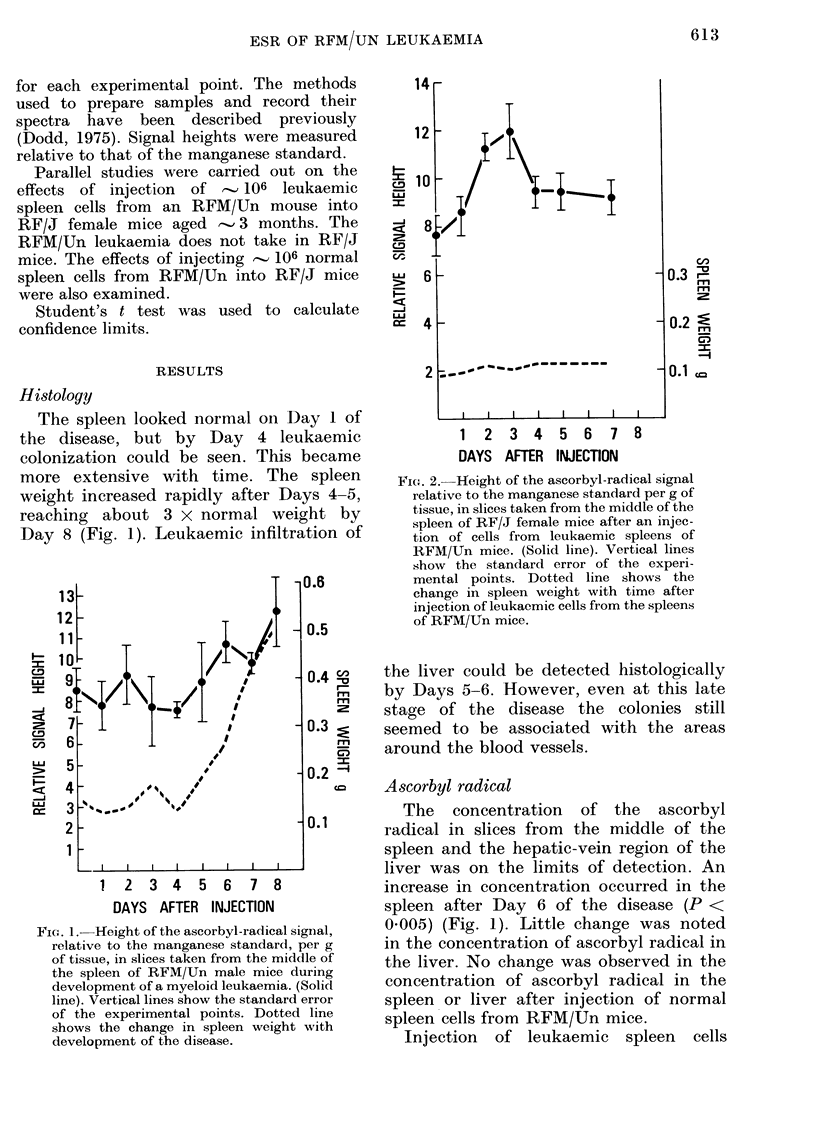

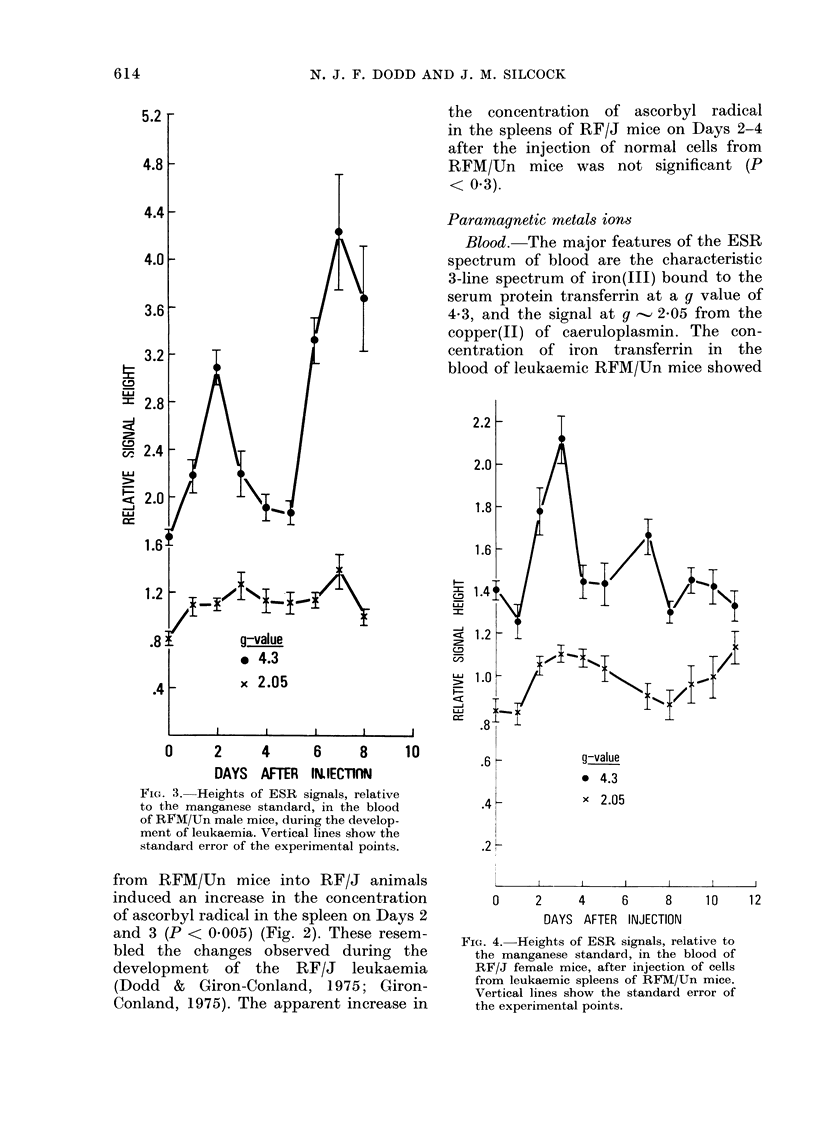

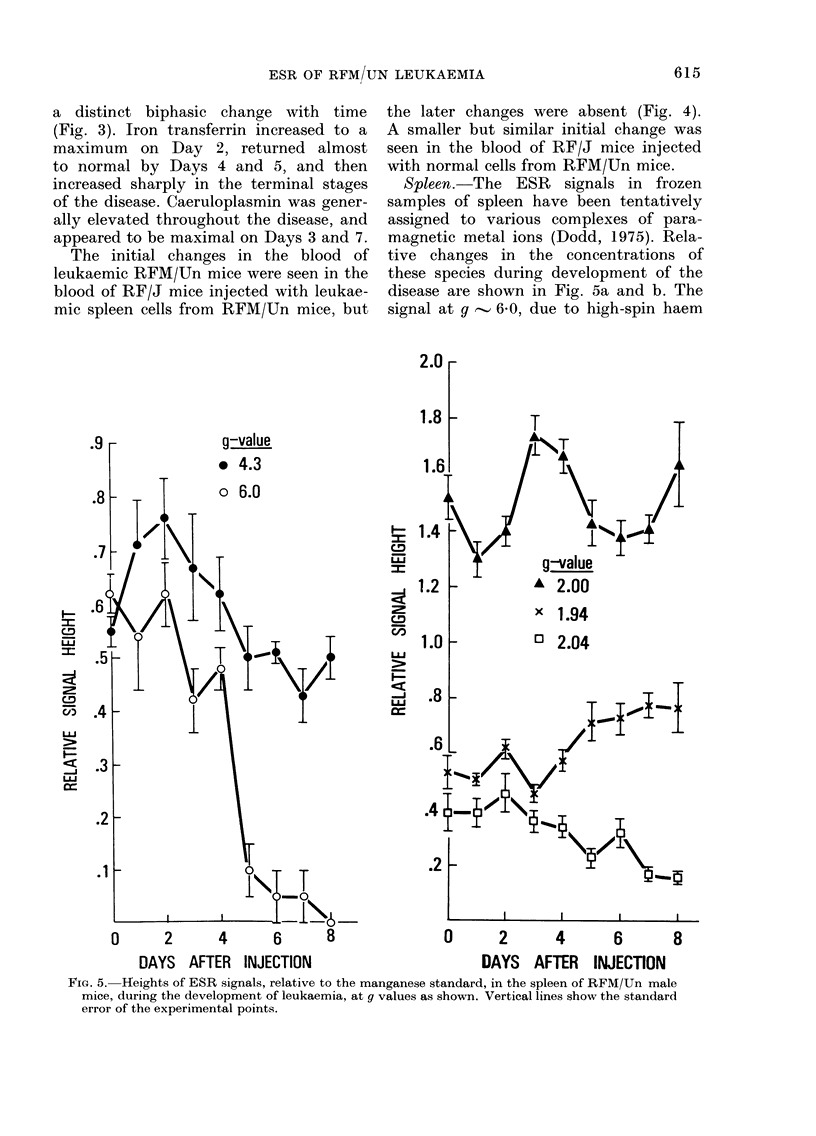

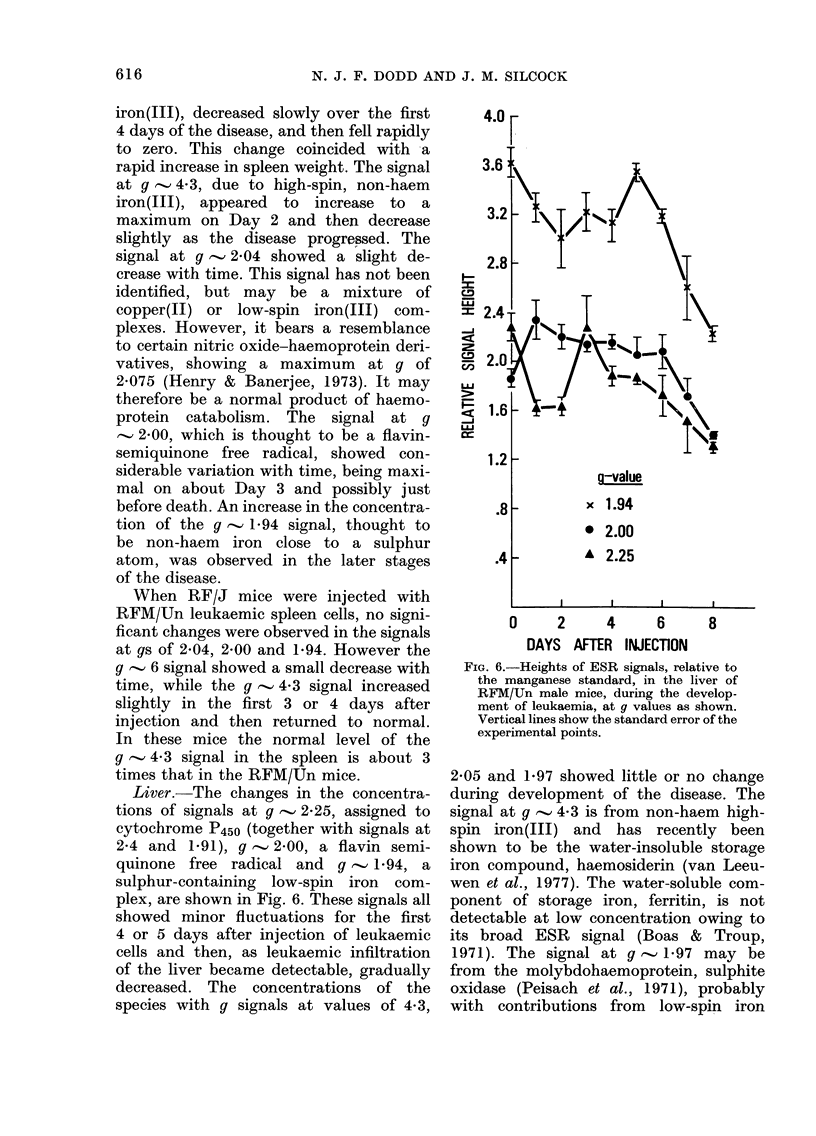

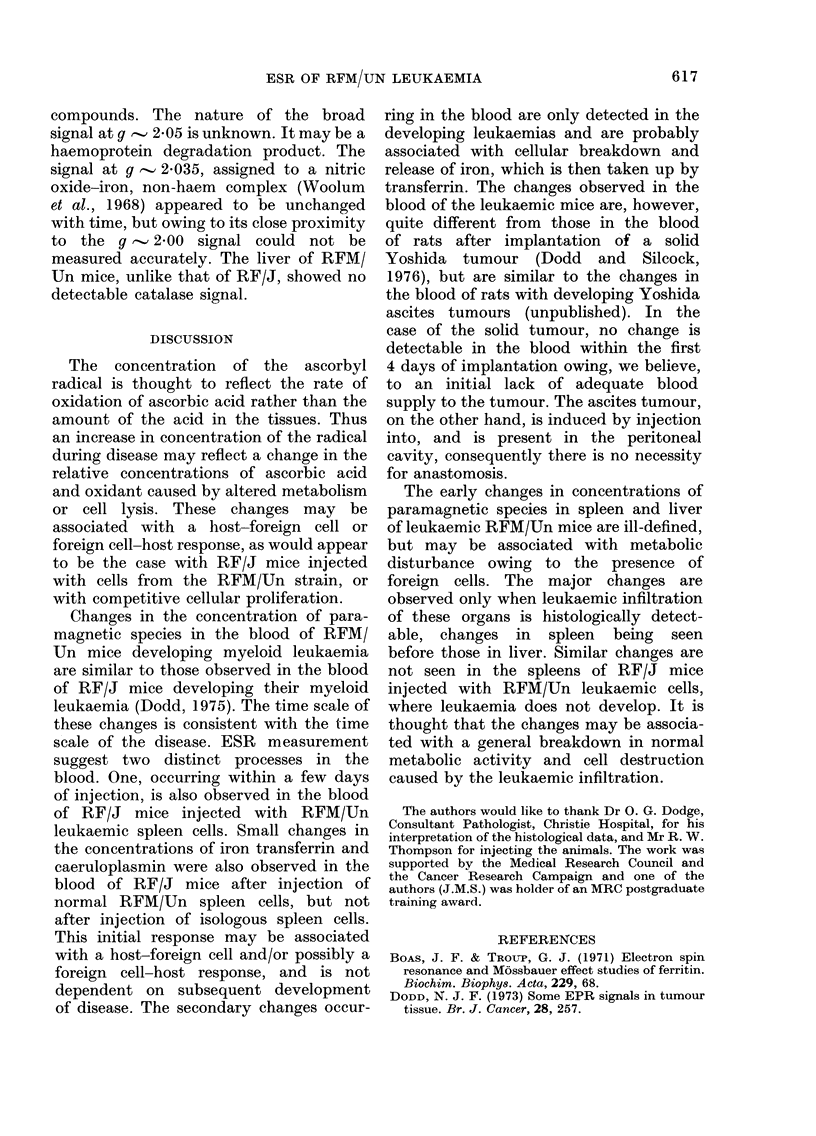

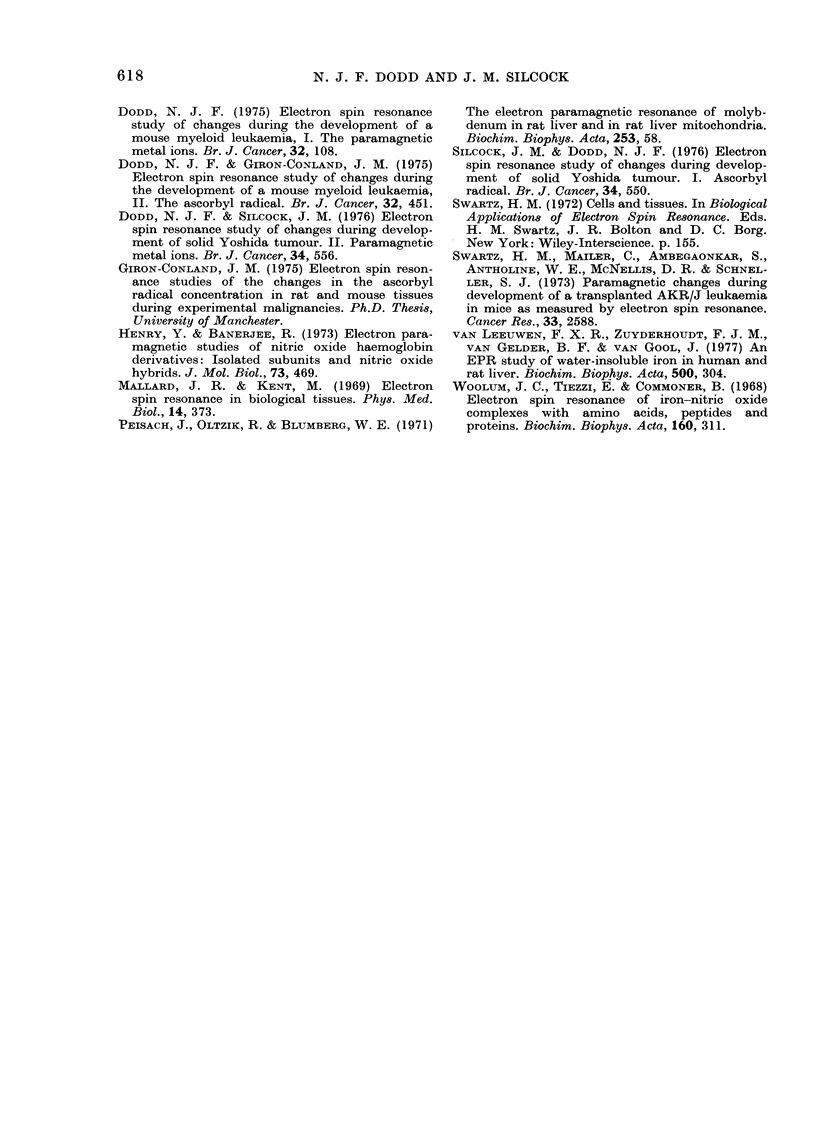


## References

[OCR_00642] Boas J. F., Troup G. J. (1971). Electron spin resonance and Mössbauer effect studies of ferritin.. Biochim Biophys Acta.

[OCR_00653] Dodd N. J. (1975). Electron spin resonance study of changes during the development of a mouse myeloid leukaemia. I. Paramagnetic metal ions.. Br J Cancer.

[OCR_00659] Dodd N. J., Giron-Conland J. M. (1975). Electron spin resonance study of changes during the development of a mouse myeloid leukaemia. II. The ascorbyl radical.. Br J Cancer.

[OCR_00664] Dodd N. J., Silcock J. M. (1976). Electron spin resonance study of changes during development of solid yoshida tumour. II. Paramagnetic metal ions.. Br J Cancer.

[OCR_00647] Dodd N. J. (1973). Some EPR signals in tumour tissue.. Br J Cancer.

[OCR_00677] Henry Y., Banerjee R. (1973). Electron paramagnetic studies of nitric oxide haemoglobin derivatives: isolated subunits and nitric oxide hybrids.. J Mol Biol.

[OCR_00683] Mallard J. R., Kent M. (1969). Electron spin resonance in biological tissues.. Phys Med Biol.

[OCR_00688] Peisach J., Oltzik R., Blumberg W. E. (1971). The electron paramagnetic resonance of molybdenum in rat liver and in rat liver mitochondria.. Biochim Biophys Acta.

[OCR_00694] Silcock J. M., Dodd N. J. (1976). Electron spin resonance study of changes during development of solid Yoshida tumour. I: Ascorbyl radical.. Br J Cancer.

[OCR_00709] Swartz H. M., Mailer C., Ambegaonkar S., Antholine W. E., McNellis D. R., Schneller S. J. (1973). Paramagnetic changes during development of a transplanted AKR-J leukemia in mice as measured by electron spin resonance.. Cancer Res.

[OCR_00720] Woolum J. C., Tiezzi E., Commoner B. (1968). Electron spin resonane of iron-nitric oxide complexes with amino acids, peptides and proteins.. Biochim Biophys Acta.

[OCR_00714] van Leeuwen F. X., Zuyderhoudt F. J., van Gelder B. F., van Gool J. (1977). Hemosiderin. an EPR study of water-insoluble iron in human and rat liver.. Biochim Biophys Acta.

